# ChatGPT Use Among Pediatric Health Care Providers: Cross-Sectional Survey Study

**DOI:** 10.2196/56797

**Published:** 2024-09-12

**Authors:** Susannah Kisvarday, Adam Yan, Julia Yarahuan, Daniel J Kats, Mondira Ray, Eugene Kim, Peter Hong, Jacob Spector, Jonathan Bickel, Chase Parsons, Naveed Rabbani, Jonathan D Hron

**Affiliations:** 1 Division of General Pediatrics Boston Children's Hospital Boston, MA United States; 2 Department of Biomedical Informatics Harvard Medical School Boston, MA United States; 3 Division of Hematology/Oncology The Hospital for Sick Kids Toronto, ON Canada; 4 Department of Pediatrics The University of Toronto Toronto, ON Canada; 5 Children's Healthcare of Atlanta Atlanta, GA United States; 6 School of Medicine Emory University Atlanta, GA United States; 7 Department of Pediatrics Harvard Medical School Boston, MA United States; 8 Atrius Health Newton, MA United States; 9 Pediatric Physicians' Organization at Children's Hospital Wellesley, MA United States; 10 Computational Health Informatics Program Boston Children's Hospital Boston, MA United States

**Keywords:** ChatGPT, machine learning, surveys and questionnaires, medical informatics applications, OpenAI, large language model, LLM, machine learning, pediatric, chatbot, artificial intelligence, AI, digital tools

## Abstract

**Background:**

The public launch of OpenAI’s ChatGPT platform generated immediate interest in the use of large language models (LLMs). Health care institutions are now grappling with establishing policies and guidelines for the use of these technologies, yet little is known about how health care providers view LLMs in medical settings. Moreover, there are no studies assessing how pediatric providers are adopting these readily accessible tools.

**Objective:**

The aim of this study was to determine how pediatric providers are currently using LLMs in their work as well as their interest in using a Health Insurance Portability and Accountability Act (HIPAA)–compliant version of ChatGPT in the future.

**Methods:**

A survey instrument consisting of structured and unstructured questions was iteratively developed by a team of informaticians from various pediatric specialties. The survey was sent via Research Electronic Data Capture (REDCap) to all Boston Children’s Hospital pediatric providers. Participation was voluntary and uncompensated, and all survey responses were anonymous.

**Results:**

Surveys were completed by 390 pediatric providers. Approximately 50% (197/390) of respondents had used an LLM; of these, almost 75% (142/197) were already using an LLM for nonclinical work and 27% (52/195) for clinical work. Providers detailed the various ways they are currently using an LLM in their clinical and nonclinical work. Only 29% (n=105) of 362 respondents indicated that ChatGPT should be used for patient care in its present state; however, 73.8% (273/368) reported they would use a HIPAA-compliant version of ChatGPT if one were available. Providers’ proposed future uses of LLMs in health care are described.

**Conclusions:**

Despite significant concerns and barriers to LLM use in health care, pediatric providers are already using LLMs at work. This study will give policy makers needed information about how providers are using LLMs clinically.

## Introduction

The public launch of ChatGPT by OpenAI in November 2022 generated immediate interest from a wide range of professionals as well as the public. The chatbot is based on a generative pretrained transformer large language model (GPT LLM) that processes large amounts of text to create an artificial intelligence (AI) system, which can then be repurposed across a variety of domains and diverse tasks [1]. Researchers and health care organizations have begun investigating how LLMs could be used and adapted within the medical field.

Some of the emerging potential applications of LLMs in medicine have included knowledge retrieval, which OpenAI has used in its own promotional material, as well as clinical decision support, medical note-taking, and composing patient communications [2-5]. However, potential problems with the application of LLMs to medicine are still not well understood. One early concern has been the phenomenon of AI “hallucinations,” in which the LLM can unpredictably return plausible sounding but incorrect or nonsensical answers to prompts [6,7]. Additionally, the content and phrasing of questions/prompts can significantly influence the LLM’s output, potentially impacting reliability [8-10]. Concerningly, the publicly available version of ChatGPT has been shown to have a diagnostic error rate of 83% in pediatric cases [11]. Other concerns regarding the clinical use of LLMs include privacy risks, lack of transparency, and bias perpetuation. In response, many institutions have begun drafting guidelines for using ChatGPT and other generative AI tools in health care settings [12].

The extent to which LLMs are already being used in health care is unclear. There is a need for better assessment of the current use practices, future intended uses, and general knowledge of health care providers regarding generative AI tools. Understanding the concerns and perspectives of clinical providers will be valuable in guiding the future development of both the AI tools themselves as well as the guidelines and principles for the use of such tools in health care.

Early surveys of ChatGPT/LLM use have focused broadly on uses beyond clinical care, have assessed perceptions rather than use, or have been limited in scope [13-17]. There is a dearth of information about health care providers’ perceptions of the benefits and barriers of LLMs in health care, and no study has assessed how pediatric providers are already using publicly available tools in their clinical and nonclinical work. This study sought to describe the knowledge and use of LLMs such as ChatGPT by physicians and advanced practice providers at a large, academic, free-standing children’s hospital.

## Methods

### Study Design

This study used a cross-sectional survey design to explore clinicians’ knowledge and use of LLMs such as ChatGPT. The survey instrument was developed using a modified Delphi method by an expert panel of 10 physician informaticians from various pediatric specialties; survey questions were collaboratively codesigned with multiple rounds of feedback until consensus was achieved. The survey consisted of a series of structured and unstructured questions and used adaptive questioning; the full survey, including the adaptive questioning logic, is available in Multimedia Appendix 1. The survey was pilot-tested on a group of 5 pediatricians prior to being sent to the entire target population.

### Ethical Considerations

The study protocol, survey, and recruitment tool were granted full approval by the Boston Children’s Hospital Institutional Review Board (#P00045317). Participation in the survey was voluntary and uncompensated; respondents were informed of the purpose of the study and assured of their anonymity. The informed consent and privacy and confidentiality protection language is provided in Multimedia Appendices 1 and 2.

### Recruitment

This closed survey study was conducted at Boston Children’s Hospital, a large academic urban pediatric health care system. The target sample was all Boston Children’s Hospital physicians and advanced practice providers in both hospital and outpatient clinic settings. Recruitment emails (see Multimedia Appendix 2) were sent via Research Electronic Data Capture (REDCap), a secure, web-based data capture application, which was also used for survey administration [18,19]. Survey recruitment and data collection started on October 12, 2023, and extended through November 14, 2023. Reminder emails were sent via REDCap to nonrespondents a maximum of 2 times.

Survey responses were analyzed in aggregate with minimum subgroup sizes of 10 responses to minimize the risk of reidentification of participants. Four survey questions included an “other” free-text option to capture concepts not covered by the provided answer choices. These free-text responses were analyzed qualitatively using the following methods. For each question, the predetermined discrete survey responses were used as a provisional codebook, which was expanded through inductive content analysis of the free-text responses [20]. To create the expanded codebook, 2 researchers (DJK and NR), one of whom was not involved with the data acquisition process, reviewed free-text responses and generated additional codes through an iterative process involving consensus meetings until no new codes were identified [21,22]. Coding conflicts were resolved by a third researcher (JDH). This expanded codebook was then applied to categorize free-text survey responses. Following coding, the 3 researchers (DJK, NR, and JDH) organized the coded responses into broader themes through an iterative process of consensus meetings.

### Subgroup Analysis

The *χ*^2^ test of independence, followed by a posthoc analysis of adjusted residuals, was used to assess differences in survey question responses across demographic variables, including age, gender, race/ethnicity, and clinical role.

## Results

### Demographic Characteristics

Surveys were sent to a total of 3127 physicians and advanced practice providers via email; we received 390 (12.5%) completed survey responses. As shown in Table 1, most respondents self-identified as female (n=293, 76.3%), White or European (n=324, 83.7%), and either an attending physician (n=165, 42.4%) or advanced practice provider (n=110, 28.3%).

**Table 1:** Characteristics of respondents who voluntarily participated in the ChatGPT/large language model survey during the survey data collection period (N=390).

Characteristic			Respondents, n (%)
**Gender**			
	Female	293 (76.3)	
	Male	85 (22.1)	
	Nonbinary	2 (0.5)	
	Prefer not to answer	4 (1.0)	
**Age (years)**			
	≤29	24 (6.2)	
	30-39	134 (36.2)	
	40-49	110 (28.3)	
	50-59	69 (17.7)	
	60-69	34 (8.7)	
	≥70	11 (2.8)	
**Race/ethnicity**			
	American Indian or Alaska Native	1 (0.3)	
	Asian or Asian American	36 (9.3)	
	Black or African American	13 (3.4)	
	Hispanic or Latino/a/e	14 (3.6)	
	Native Hawaiian or Pacific Islander	0 (0)	
	White or European	324 (83.7)	
	Something else	4 (1.0)	
	Prefer not to say	12 (3.1)	
**Role**			
	Attending physician	165 (42.4)	
	Advanced practice provider	110 (28.3)	
	Resident/fellow	32 (8.2)	
	Other	82 (21.1)	
**Specialty**			
	Anesthesia	14 (3.6)	
	Emergency medicine	20 (5.1)	
	General pediatrics	59 (15.2)	
	Pediatric subspecialty	149 (38.3)	
	Radiology	7 (1.8)	
	Pathology	2 (0.5)	
	Surgical specialty	50 (12.9)	
	Other	88 (22.6)	

### Familiarity With and Current Use of ChatGPT

Among the 390 respondents, 288 (73.7%) indicated that they were familiar with ChatGPT or another LLM and an additional 83 (21.2%) indicated that they had heard of ChatGPT but did not really know what it is. Only 19 (4.9%) respondents reported that they had not heard of ChatGPT. Of those who had heard of ChatGPT (n=371), 197 (53.1%) had used ChatGPT or a similar model. Only 52 (26.7%) of 195 respondents who were using an LLM had used it clinically. Reported clinical uses of LLMs are shown in Table 2; the most common uses were drafting school and work letters and drafting prior authorizations.

Overall, 72.4% (142/196) of question respondents reported using LLMs for nonclinical work. Nonclinical use cases included drafting emails (55/139 39.6%); creating outlines for grants, papers, or teaching materials (52/139, 37.4%); drafting a letter of recommendation (51/139, 36.7%); and writing code (eg, for statistical analysis or data visualization) (18/139 12.9%). Respondent free-text uses that were listed in “other” are shown in Table 3.

Most respondents did not think ChatGPT should be used for patient care in its present state (256/390, 70.9%). Listed concerns were accuracy or reliability (319/390, 87.2%), patient privacy or security (237/390, 64.8%), unclear how ChatGPT makes decisions (232/390, 63.4%), lack of regulation (225/390, 61.5%), and potential bias in the data model (219/390, 59.8%). Free-text responses for this question are shown in Table 3.

**Table 2:** Clinical use cases endorsed by survey respondents who indicated that they had used ChatGPT or a large language model (LLM) in their clinical work (n=52).

Responses to “How have you used ChatGPT/LLM in your clinical work (select all that apply)?”	Respondents, n (%)
Draft school or work letter	26 (50)
Draft prior authorization	18 (35)
Generate patient education materials	15 (29)
Generate differential diagnosis	12 (23)
Other	12 (23)
Ask a specific clinical question (not mentioned above)	11 (21)
Draft all or part of a clinical note	8 (15)
Suggest a treatment plan	8 (15)
Respond to patient inbox messages	5 (10)
Draft all or part of a discharge summary	3 (6)
Draft handoff documentation	3 (6)

**Table 3:** Free-text responses collected for 4 questions regarding participant use of and beliefs about large language models in clinical and nonclinical work.

Coded survey response^a^			Respondents, n		Example
**How have you used ChatGPT to help you with your non-clinical work?**					
	Information search	12		It is a helpful adjunct to online searching. Helps you quickly narrow what you are looking for (assuming you don't want a broad search)	
	Plan recreational activity	8		Used for coming up with ideas for nonwork-related group activities	
	Summarize text	5		Summarize review papers into usable notes.	
	Revise communication	5		Editing text for grammar	
	Literature review	3		Triage/screen PubMed abstracts to identify references of interest	
	Generate title	3		Generate catchy titles for manuscripts and presentations.	
	Ideation	3		Generate research ideas	
	Draft mass communication	3		social media for my business	
	Creative writing	2		Write poems (in English and other languages), generate ideas	
	Well-being programming	2		Generate relaxation scripts.	
	Workflow	1		Write workflow proposals	
	Draft cover letter	1		Write … cover letters	
	Translation	1		translation of materials from English to another language	
	Task management	1		organizing to-do lists	
**What concerns do you have about using ChatGPT clinically?**					
	Perceived lack of utility	5		Still not clear on how it would be used in healthcare	
	Potential bias in the data model	3		At times, ChatGPT is confounded by the presence of wrong data and, therefore, presents clearly inaccurate statements.	
	Legal	3		legal concerns- I am so careful about my documentation, and I just don't think chat GPT will ever word things the way I need it to help me in medicolegal situations.	
	Automation bias	2		Worried about clinician interpretation of ChatGPT output … and cannot replace clinical reasoning	
	Plagiarism	2		It is plagiarism on steroids.	
	Learning curve	1		Would not like to have to master a new technology in addition to the onslaught of requests for computer interface as it is	
	Depersonalization	1		That it could take away from collaborative development of an illness explanation that provider and patient/family engage in together.	
	Skill atrophy	1		Humans writing reports allows clinicians to integrate data in a way that supports clinical decision making and patient counseling. I am already finding a lack of critical thinking skills in graduate students. Push button documentation would be efficient (and report writing is arduous) but we all still need to think.	
**How would you use the Boston Children’s Hospital HIPAA^b^-compliant version of ChatGPT if it were available?**					
	Research	6		My AI robot will ... learn to sort data in redcap	
	Translation	3		I use it probably in the most simple of ways to translate patient handouts into their language.	
	Summarize clinical narrative	3		Summarization of complex patient medical history and relevant clinical information and other data aggregation tasks (e.g., ascertain primary/longitudinal care team members involved in patient's care)	
	Extract data from narratives	1		Review patient charts and imaging reports to generate tabular data for research.	
	Workflow	1		Lots of potential nonclinical purposes, describing workflow, responsibility mapping	
**If a HIPAA-compliant version of ChatGPT were available, what types of information would you feel comfortable entering?**					
	Demographic data	1		Name of patient’s school	
	Patient medications	1		Info related creating a prior auth insurance, medication, dx, etc.	

^a^The responses were analyzed and codified by a team of 3 physicians using formal qualitative methodology.

^b^HIPAA: Health Insurance Portability and Accountability Act.

### Future Use of ChatGPT

Among the 367 respondents, 272 (74.1%) indicated they would use a version of ChatGPT if one were available that was compliant with the Health Insurance Portability and Accountability Act (HIPAA) [23]. Table 4 shows examples of how they envisioned using a HIPAA-compliant version of ChatGPT. If such a model were available, most participants indicated that they would feel comfortable entering patient diagnoses (223/266, 83.9%), age (188/266, 70.7%), and clinical questions without patient information (186/266, 69.9%); few would feel comfortable entering patient name (101/266, 38.0%), medical record number (87/266, 33%), date of birth (98/266, 37%), or whole notes from a patient chart (99/266, 37%). Table 3 shows the results of our analysis for free-text responses.

**Table 4:** Clinical use cases envisioned by pediatric providers who responded to the survey question about how they would use a Health Insurance Portability and Accountability Act (HIPAA)–compliant version of ChatGPT if one were available (n=270).

Responses to “How would you use a HIPAA compliant version of ChatGPT (select all that apply)?”	Respondents, n (%)
Draft school or work letter	209 (77)
Generate patient education materials	191 (71)
Draft all or part of a clinical note	169 (63)
Draft prior authorization	159 (59)
Ask ChatGPT a specific clinical question	114 (42)
Draft all or part of a discharge summary	104 (39)
Generate differential diagnosis	99 (37)
Respond to patient inbox messages	87 (32)
Suggest a treatment plan	76 (28)
Draft handoff documentation	72 (27)
Other	23 (9)

### Subgroup Analysis

Trainees were more likely to have used ChatGPT than other respondents (*P*=.01); the percentage who endorsed using ChatGPT clinically was higher among trainees (40%) than nontrainees (24%), but the values in this question did not reach statistical significance (*P*=.06). Male respondents were also more likely to have used ChatGPT (*P*=.005). Respondents in the ≤29 years age group were more likely to be familiar with ChatGPT and those in the ≥70 years age group were less likely (*P*=.002). Responses to the rest of the survey questions did not significantly differ across demographics. Specifically, there was no statistically significant difference in whether an LLM was being used for clinical and/or nonclinical work, the endorsed current use cases, expressed concerns regarding LLM use in health care, desire for a HIPAA-compliant LLM, or endorsed planned uses for a HIPAA-compliant LLM.

## Discussion

### Principal Findings

This study demonstrates that pediatric providers are already using LLMs in both their clinical and nonclinical work. Because of the known limitations of LLMs in a clinical setting, including demonstrated low diagnostic accuracy, it is important to know how pediatricians are using these tools. This study adds to the current literature by providing granular information about how people are currently using LLMs in their work as well as detailing ways that providers envision using a HIPAA-compliant future version of ChatGPT.

Nearly all survey respondents had heard of ChatGPT, and most had used this tool. While nearly 75% of LLM users indicated that they are already using ChatGPT in their nonclinical work, only slightly over 25% indicated that they are currently using an LLM for clinical work. Moreover, the most common clinical uses reported were for administrative tasks such as drafting letters, prior authorizations, and patient education materials.

Similar to other early studies, we found that clinicians are enthusiastic about using LLMs (specifically, a HIPAA-compliant LLM) in clinical and nonclinical work. It is notable that almost one-third of pediatric providers in this study indicated that they would feel comfortable using LLMs for patient care in the current format. Additionally, almost three-quarters of respondents indicated that they would use a HIPAA-compliant version of ChatGPT if one were available. If a HIPAA-compliant LLM were available, participants described a variety of ways that they would use it. Most of the envisioned use cases for the HIPAA-compliant LLM were still administrative or operational; however, other common uses included drafting all or part of a clinical note as well as a variety of uses related to clinical decision support and clinical documentation. 

Since we have demonstrated that LLMs are already being used clinically and that there is strong interest in further future use of LLMs, it is imperative that health care systems and government agencies create thoughtful policies and regulations for LLM use in health care. The Biden administration recently announced an executive order that directs actions to maximize the promise and manage the risks of AI [24]. Clinical informaticians are needed to help navigate the thoughtful implementation of AI tools into clinical care.

### Limitations and Future Work

This study has limitations. Most notably, the generalizability is limited by the low response rate and pediatric provider population. Another limitation is selection bias, as providers using LLMs may be more interested in completing a survey related to this topic. Similarly, although recent American Board of Pediatric statistics show that 67% of pediatricians are female and 57% are White [25], our survey respondents were even more skewed toward these populations. Self-reported data may contain some social desirability bias as respondents may attempt to demonstrate that they are using these technologies in acceptable ways. Also of note, we did not ask if respondents worked in an inpatient, outpatient, or other setting; not having that information limits some interpretation of the response data. For example, the number of providers interested in using LLMs to write discharge summaries is less interpretable as these documents are not generally written by outpatient providers.

The enthusiasm we found for the future use of LLMs lends itself to further investigation of LLM use in health care. We propose evaluating the differences in use cases by clinical work setting such as the emergency department versus inpatient versus outpatient versus proceduralists. There would be value in determining if survey results would differ across practice settings such as nonacademic, nonurban, adult patients, and different patient resources, or by geographic location; thus, we propose a larger study across institutions in the future. In-depth interviews and other qualitative methods could be used to gain deeper insights into providers’ LLM use and beliefs. Finally, exploring patients’ perceptions and current use of LLMs would be of great value.

### Conclusions

This survey study adds to the corpus of knowledge on how providers are thinking about and using LLMs in a clinical context. LLMs will add to the series of digital tools in the clinical ecosystem meant to help advance clinical care. Despite significant concerns and barriers to LLM use in health care, this survey demonstrates that these tools are already commonly used and there is enthusiasm for their future use. Knowing how providers are using LLMs in their clinical and nonclinical work will help guide policy and regulations regarding the health care use of AI. As informaticians, it is incumbent upon us to support the appropriate use of these technologies to improve patient care, while also monitoring for their unintended consequences.

## Appendix

Multimedia Appendix 1 Survey questions.

Multimedia Appendix 2 ChatGPT survey recruitment email.

## Abbreviations

AI: artificial intelligence
GPT LLM: generative pretrained transformer large language model
HIPAA: Health Insurance Portability and Accountability Act
LLM: large language model
REDCap: Research Electronic Data Capture

## Data Availability

The datasets generated and analyzed during this study are available from the corresponding author on reasonable request.
