# Supplementary material for: ChatGPT Use Among Pediatric Health Care Providers: Cross-Sectional Survey Study
Source: JMIR Form Res. 2024 Sep 12;8:e56797. doi: 10.2196/56797 (PMC11427860; doi:10.2196/56797)
Supplement: Multimedia Appendix 1 [file formative_v8i1e56797_app1.docx]

Multimedia Appendix 1

This appendix provides the exact language and adaptive questioning logic of the ChatGPT/LLM survey conducted at Boston Children's Hospital in October and November of 2023. Participants were able to change any answers prior to submission and a back button was available to return to previously answered questions.

ChatGPT and other Large Language Model Use Survey

**Please complete the survey below**

Thank you!

Introduction and Disclosure:

To better understand current and desired future use of large language models (LLMs) like ChatGPT at Boston Children’s Hospital, we encourage you to complete this voluntary survey. We have invited all physicians and advanced practice providers to complete this survey. Your participation in this survey is completely voluntary and the data will be stored in a de-identified and secured manner; few people will be able to access your answers and no one will be able to connect you with your answers. Data, results, and reports related to this survey will be maintained securely and under privilege. If you do not wish to participate in this study, you may simply close this browser window. There is no penalty for not completing this survey. Continuing with this survey will be accepted as your consent to participate in the survey.

PART A DEMOGRAPHICS

A1) What is your role at Boston Children’s Hospital:

[ ] Attending MD

[ ] Advanced Practice Provider

[ ] Fellow

[ ] Resident

[ ] Other

A2) If selection attending or fellow branch to list of specialties

Which of these best describes you?

[ ] Anesthesia

[ ] Emergency Medicine

[ ] General Pediatrics

[ ] Pediatric Subspecialty (ex. Endocrinology, Critical Care)

[ ] Radiology

[ ] Surgical specialty (ex. Urology, ORL)

[ ] Other: (free text)

Free text box says: What specialty would best describe your role at BCH?

A3) What gender do you most associate with?

[ ] Female

[ ] Male

[ ] Non-binary

[ ] Other

[ ] Prefer not to answer

A4) Race/ethnicity identification (Check all that apply.)

[ ] American Indian or Alaska Native

[ ] Asian or Asian American

[ ] Black or African American

[ ] Hispanic or Latino/a/e

[ ] Native Hawaiian or Pacific Islander

[ ] White or European

[ ] Something else

[ ] Prefer not to answer

If a respondant chooses, “something else,” 🡪 A5

Otherwise, 🡪A6

A5) My race or ethnicity is best described as:

[ ] Free text

A6) What is your current age?

[ ] ≤ 29

[ ] 30-39

[ ] 40-49

[ ] 50-59

[ ] 60-69

[ ] ≥ 70

PART B HISTORIC USE OF CHATGPT FOR CLINICAL WORK

B1) Have you heard of ChatGPT and/or other large language models (LLMs)?

[ ] Yes; I am familiar with ChatGPT and/or other LLMs

[ ] Yes; I’ve heard of ChatGPT but don’t really know what it is

[ ] No

* If answer is no, end survey.

ChatGPT is a large language model developed by OpenAI based on the GPT (Generative Pre-trained Transformer) architecture. It is a form of artificial intelligence built to understand natural language input and generate responses in order to participate in human-like conversation.  ChatGPT is trained on a vast volume of data including text from various sources such as books, articles, and websites, which enables interaction on a wide range of topics, including health care. Other large language models and software that leverage generative AI technology include Bing, Google’s Bard, Meta’s LLaMa and others.

B2) Have you used ChatGPT or a similar model?

[ ] Yes

[ ] No

*if answer is yes -> B3

*if answer no -> skip to Part B7

ChatGPT and other LLMs may be useful clinical tools. Existing versions of ChatGPT and LLMs are not HIPAA-compliant; entering protected health information in this type of program violates current hospital policy. We are interested in how you may be currently using LLMs in your clinical work.

B3) Have you used ChatGPT or a similar model to help you with your clinical work?

[ ] Yes

[ ] No

*if answer is yes -> B4

*if answer no -> skip to B5

B4) How have you used ChatGPT/LLM to help you with your clinical work (select all that apply)?

[ ] Draft all or part of a clinical note

[ ] Draft all or part of a discharge summary

[ ] Draft handoff documentation

[ ] Draft prior authorization

[ ] Draft school / work letter

[ ] Generate differential diagnosis

[ ] Suggest a treatment plan

[ ] Respond to patient inbox messages

[ ] Generate patient education materials

[ ] Ask a specific clinical question (not mentioned above)

[ ] Other

B5) Have you used ChatGPT or a similar model to help you with non-clinical work activities?

[ ] Yes

[ ] No

*if answer is yes -> B6

*if answer no -> skip to Part B7

B6) How have you used ChatGPT to help you with your non-clinical work (select all that apply)?

[ ] Draft emails

[ ] Create outline for grants/papers/teaching

[ ] Draft letter of recommendation

[ ] Write code (e.g. for statistical analysis or data visualization)

[ ] Other: ____ (free text)

B7) What concerns do you have about using ChatGPT clinically?

[ ] Concerns about accuracy or reliability

[ ] Unclear how ChatGPT makes decisions

[ ] Concern about patient privacy or security

[ ] Concern about lack of regulation

[ ] Potential bias in the data model

[ ] Other: ________________________ (free text)

PART C FUTURE USE OF CHATGPT

C1) Do you think ChatGPT in its present state should be used for patient care?

[ ] Yes

[ ] No

C2) If Boston Children’s Hospital had a HIPAA compliant version of ChatGPT where protected health information (PHI) could be securely entered would you use it?

[ ] Yes

[ ] No

* If answer to C2 is yes --> C3, C4, C5

* If no, --> C5

C3) How would you use the Boston Children’s Hospital HIPAA-compliant version of ChatGPT if it were available?

[ ] Draft all or part of a clinical note

[ ] Draft all or part of a discharge summary

[ ] Draft handoff documentation

[ ] Draft prior authorization

[ ] Draft school / work letter

[ ] Generate differential diagnosis

[ ] Suggest a treatment plan

[ ] Ask ChatGPT specific clinical questions

[ ] Respond to patient inbox messages

[ ] Generate patient education materials

[ ] Other: ____ (free text)

C4) If a HIPAA-compliant version of ChatGPT were available, what types of information would you feel comfortable entering?

[ ] Patient’s name

[ ] Patient’s MRN

[ ] Patient’s date of birth

[ ] Patient’s age

[ ] Patient’s diagnosis

[ ] Whole notes from patient chart

[ ] Clinical question with no patient information

[ ] Other: ____ (free text)
