# Supplementary material for: ChatGPT Use Among Pediatric Health Care Providers: Cross-Sectional Survey Study
Source: JMIR Form Res. 2024 Sep 12;8:e56797. doi: 10.2196/56797 (PMC11427860; doi:10.2196/56797)
Supplement: Multimedia Appendix 2 [file formative_v8i1e56797_app2.docx]

Multimedia Appendix 2

Dear Clinicians,

There is increasing interest in the use of artificial intelligence in healthcare for both clinical and administrative purposes.  Since ChatGPT and other large language models have become publicly available, many people are already using these tools in their daily lives.  This study aims to explore clinicians' current knowledge and use of artificial intelligence tools like ChatGPT and to help guide future decisions about the appropriate use of these technologies in healthcare.

Please complete this brief survey to help us better understand current and future uses of artificial intelligence tools for clinical care and other health care administrative activities.  The survey will take less than**3-5 minutes** or less to complete, but you must be on the Boston Children's network or BCH VPN .

This is a voluntary survey, and all responses are anonymous. This study has been approved by BCH IRB.  The survey is hosted on REDCap, a secure data transmission platform.  No one will be able to connect your responses to you. This information is intended to inform the possible use of ChatGPT and other Large Language Models at BCH and is intended for journal publication.  Any possible future publication will not have any individual identification.

- The RedCap survey is designed so that we cannot link precise answers to you.
- Reporting of summary data will not use small bin sizes which should further limit any ability to identify specific providers.

Clicking on the links below indicate your consent to take part in this study and the use of your anonymous responses.

The survey only works if you are on the Boston Children's network or BCH VPN.  You may open the survey in your web browser by clicking the link below:
[USE THE LINK YOU RECEIVED IN YOUR EMAIL]

If you are on the BCH network or VPN and the link above does not work, try copying the link below into your web browser:
https://redcap-qi.tch.harvard.edu/redcap_edc/surveys/?s=zoi7x8RczEC8j2CC

This link is unique to you and should not be forwarded to others.

 Please reach out to[Jonathan.Hron@childrens.harvard.edu](mailto:Jonathan.Hron@childrens.harvard.edu) or [Susannah.Kisvarday@childrens.harvard.edu](mailto:Susannah.Kisvarday@childrens.harvard.edu) if you have any questions.

Thank you in advance for your participation!
